# Optimal Hemoglobin A1c Levels for Screening of Diabetes and Prediabetes in the Japanese Population

**DOI:** 10.1155/2015/932057

**Published:** 2015-05-31

**Authors:** Masanori Shimodaira, Shinji Okaniwa, Norinao Hanyu, Tomohiro Nakayama

**Affiliations:** ^1^Department of Internal Medicine, Iida Municipal Hospital, 438 Yawata-machi, Iida, Nagano 395-8502, Japan; ^2^Division of Companion Diagnostics, Department of Pathology of Microbiology, Nihon University School of Medicine, 30-1 Ooyaguchi-kamimachi, Itabashi-ku, Tokyo 173-8610, Japan

## Abstract

The aim of this study was to evaluate the utility of hemoglobin A1c (HbA1c) to identify individuals with diabetes and prediabetes in the Japanese population. A total of 1372 individuals without known diabetes were selected for this study. A 75 g oral glucose tolerance test (OGTT) was used to diagnose diabetes and prediabetes. The ability of HbA1c to detect diabetes and prediabetes was investigated using receiver operating characteristic (ROC) analysis. The kappa (*κ*) coefficient was used to test the agreement between HbA1c categorization and OGTT-based diagnosis. ROC analysis demonstrated that HbA1c was a good test to identify diabetes and prediabetes, with areas under the curve of 0.918 and 0.714, respectively. Optimal HbA1c cutoffs for diagnosing diabetes and prediabetes were 6.0% (sensitivity 83.7%, specificity 87.6%) and 5.7% (sensitivity 60.6%, specificity 72.1%), respectively, although the cutoff for prediabetes showed low accuracy (67.6%) and a high false-negative rate (39.4%). Agreement between HbA1c categorization and OGTT-based diagnosis was low in diabetes (*κ* = 0.399) and prediabetes (*κ* = 0.324). In Japanese subjects, the HbA1c cutoff of 6.0% had appropriate sensitivity and specificity for diabetes screening, whereas the cutoff of 5.7% had modest sensitivity and specificity in identifying prediabetes. Thus, HbA1c may be inadequate as a screening tool for prediabetes.

## 1. Introduction

Diabetes is reaching pandemic proportions across the world. The number of diabetes patients is estimated to increase from 8.3% (366 million) globally in 2011 to 9.9% (552 million) by 2030 [1]. Prediabetes usually precedes diabetes and is more prevalent than diabetes [2]. Even in the prediabetic state, micro- and macrovascular complications have been reported [3]. Therefore, efficient identification of subjects with diabetes and prediabetes is paramount to prevent complications or delay disease progression from prediabetes to diabetes.

Fasting plasma glucose (FPG) and 2 h postload plasma glucose (2h-PG) during a 75 g oral glucose tolerance test (OGTT) have traditionally been used as the gold standard tests for diagnosing diabetes and prediabetes. However, both tests require a fasting state, which is an important barrier to screening. Furthermore, the reproducibility of 2h-PG and concordance between the FPG and 2h-PG levels were poor [4, 5]. An International Expert Committee (IEC) recently proposed new diagnostic criteria on the basis of the HbA1c measurement, with HbA1c > 6.5% being definitive for diabetes and the range 6.0–6.4% as indicative of “high risk” of progression to diabetes [6]. In 2010, the American Diabetes Association (ADA) recommended an alternate HbA1c cutoff of 5.7–6.4% for prediabetes, designated as a “category of increased risk for diabetes” [7]. The IEC stated that HbA1c “may be better” than other glycemic criteria for diagnosing diabetes [6], and the ADA listed the HbA1c criteria as “superior to” the glycemic criteria [7].

HbA1c is relatively well standardized, exhibits low intraindividual variation, and does not require fasting or restriction to certain times of the day; therefore, the use of HbA1c measurements to screen patients for diabetes and prediabetes will likely be popular with clinicians. However, variations in HbA1c as a function of glucose-independent factors such as race and ethnicity have been reported [8–10]. The HbA1c cutoffs for diagnosing diabetes and prediabetes in the Japanese population are unclear. The aim of this study was to identify optimal HbA1c cutoff values for the diagnosis of diabetes and prediabetes and to evaluate the utility of HbA1c as a screening tool for these glucose disorders in the general Japanese population.

## 2. Materials and Methods

### 2.1. Study Participants

Between January 2008 and December 2014, 1372 individuals (≥35 years) with no known history of diabetes underwent the 75 g OGTT as part of a routine health examination at Iida Municipal Hospital, Iida, Nagano, Japan. Participants were required to fast for at least 10 hours overnight, avoid heavy physical activity on the day before examination, and refrain from smoking before and during the test.

### 2.2. Definition of Diabetes and Prediabetes

On the basis of the 2014 ADA criteria, normal glucose tolerance was defined as a FPG of <100 mg/dL and a 2h-PG of <140 mg/dL. Prediabetes was defined as a FPG of 100–125 mg/dL and/or 2h-PG of 140–199 mg/dL. Diabetes was defined as a FPG ≥ 126 mg/dL and/or 2h-PG ≥ 200 mg/dL [11].

### 2.3. Laboratory Assays

Serum total cholesterol, triglycerides, high-density lipoprotein cholesterol, low-density lipoprotein cholesterol, uric acid, and creatinine were determined with standard enzymatic spectrophotometric techniques (Hitachi 747 automatic analyzer, Hitachi, Ibaraki, Japan) in the hospital. Estimated glomerular filtration rates (eGFR) were calculated using the formula of the Japanese Society of Nephrology as follows: 194 × serum creatinine^−1.094^  × age^−0.287^ mL/min/1.73 m^2^, further multiplied by 0.739 for female subjects [12]. All plasma glucose and HbA1c samples were evaluated in the same laboratory. Plasma glucose samples collected in fluoride oxalate tubes were assessed within 2 h of sampling using a hexokinase method (GLU-HK, Shinotest Inc., Kanagawa, Japan), which has an imprecision coefficient of variation of 1.61%. HbA1c was measured on the same day with an ion-exchange high-performance liquid chromatography method (ARKRAY HA-8170, ARKRAY Inc., Tokyo, Japan). The imprecision coefficient of variation of this instrument is 0.96% and 1.56% at HbA1c levels of 5.8% and 7.0%, respectively, which are within the most stringent requirements of 2% [13].

### 2.4. Statistical Analysis

Statistical analyses were performed using the SPSS software version 21.0 (SPSS Inc., Chicago, IL, USA). One-way ANOVA was used to compare groups for continuous variables and a chi-square test was used for categorical variables. ROC curves were plotted and the area under the curve (AUC) of ROC was calculated for HbA1c; an AUC value of <0.7 was considered suboptimal. The optimal cutoff values for HbA1c for detecting diabetes and prediabetes (OGTT-based diagnoses) were identified using the maximum of the Youden index [(sensitivity + specificity) − 1] [14]. We assessed sensitivity, specificity, positive and negative predictive values, positive and negative likelihood ratios, and diagnostic accuracy at various HbA1c thresholds. The kappa (*κ*) coefficients were used to test for agreement between HbA1c categorization and OGTT-based diagnoses. *κ* coefficients between 0.8 and 1.0 are interpreted as indicating an almost perfect agreement, and those between 0.6 and 0.8 are indicative of substantial agreement [15]. Confidence intervals (CI) were set at 95%, and *p* < 0.05 was considered statistically significant.

## 3. Results

Table 1 shows the clinical characteristics of subjects diagnosed on the basis of the 75 g OGTT. The prevalence of prediabetes and diabetes in the study population was 36.6% and 6.3%, respectively. The frequency of subjects aged ≥ 60 years was higher in the prediabetes and diabetes groups compared with the normal glucose tolerance group. HbA1c levels (%) were 5.4 ± 0.3, 5.7 ± 0.6, and 6.7 ± 1.2 in individuals diagnosed as having normal glucose tolerance (*n* = 784), prediabetes (*n* = 502), and diabetes (*n* = 86), respectively. Compared to subjects with normal glucose tolerance, subjects with prediabetes and diabetes were more likely to be older and have a higher BMI, higher blood pressure, elevated triglycerides and uric acid, and lower high-density lipoprotein cholesterol (all *p* values <0.001).

**Figure 1:**
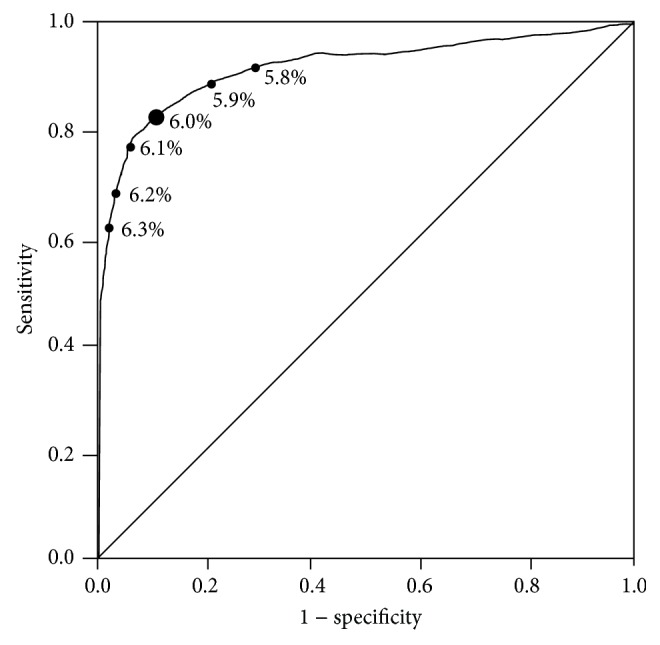
ROC curve analysis for the ability of HbA1c to predict diabetes defined by OGTT values.

**Figure 2:**
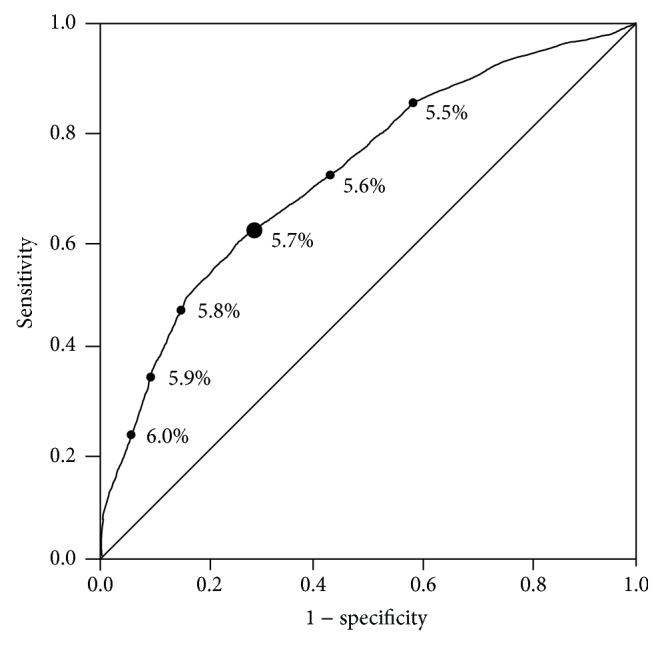
ROC curve analysis for the ability of HbA1c to predict prediabetes defined by OGTT values.

AUCs of ROC analysis of HbA1c for the diagnosis of diabetes and prediabetes were 0.918 (95% CI, 0.879–0.958) and 0.714 (95% CI, 0.685–0.743), respectively (Figures 1 and 2). The sensitivity decreased, whereas the specificity increased as the cutoff levels increased. In the analysis stratified by age, the AUCs of ROC were approximately 0.9 for diabetes and 0.7 for prediabetes (Table 2). The optimal HbA1c cutoff levels as identified by the maximal Youden index were 6.0% for diabetes and 5.7% for prediabetes. In diabetes, the HbA1c cutoff of 6.0% showed high sensitivity (83.7%) and specificity (87.6%), with a low proportion (16.3%) of false-negative results in disease identification (Table 3). In contrast, in prediabetes, the HbA1c cutoff of 5.7% had modest sensitivity (60.6%) and specificity (72.1%), with a high proportion (39.4%) of false-negative results (Table 4). At these HbA1c cutoffs (6.0% for diabetes and 5.7% for prediabetes), accuracy was high (87.4%) for diabetes and low (67.6%) for prediabetes; however, the agreement between HbA1c categorization and OGTT-based diagnoses was low in both diabetes (*κ* = 0.399) and prediabetes (*κ* = 0.324).

## 4. Discussion

The HbA1c test has recently been recommended for the diagnosis of diabetes on the basis of a detailed analysis of IEC [6] and ADA recommendations [16]. Although IEC and ADA do not recommend the use of their criteria for screening, there are several advantages of using the HbA1c test to screen patients for abnormal glucose metabolism as follows: (1) HbA1c does not require a fasting state prior to testing; (2) HbA1c reflects longer term glycemia than plasma glucose; (3) the laboratory methods evaluating HbA1c are now well standardized and reliable; and (4) errors caused by nonglycemic factors affecting HbA1c, such as hemoglobinopathies, are infrequent [17]. It is important to identify individuals with abnormal glucose metabolism, as early intervention could prevent or delay the onset of complications related to hyperglycemia.

In previous studies, the optimal HbA1c cutoffs for detecting undiagnosed diabetes ranged from 5.3% to 6.1% in population-based studies among Australian [18] and American populations [19]. Among the Hong Kong Chinese, an optimal HbA1c cutoff point of 6.1% for detecting 2h-PG ≥ 200 mg/dL was reported, which had a sensitivity of 77.5% and a specificity of 78.8% [20]. These differing results may be attributable to differences in the internal and external environments of blood cells, ethnic differences in HbA1c genes, different metabolic pathways, or other factors [8–10]. In the present study, we evaluated the optimal HbA1c cutoffs for the diagnosis of prediabetes and diabetes in the Japanese general population. The ROC curve analysis indicated the good performance of HbA1c in the identification of diabetes and prediabetes, as defined by OGTT-based diagnoses.

IEC [6] and ADA [16] have proposed new diagnostic criteria for diabetes on the basis of HbA1c measurement, in which HbA1c of ≥6.5% is defined as diabetes. In the present study, an HbA1c level of 6.5% had a reasonably high specificity (99.1%) and low false-positive rate (0.9%) for the diagnosis of diabetes, in complete concordance with IEC and ADA recommendations. However, we observed that an HbA1c of 6.5% has low sensitivity (53.5%) when used as a threshold for the identification of diabetes. In contrast, an HbA1c cutoff of 6.0% predicts diabetes with an ideal sensitivity (83.7%) and specificity (87.6%). Recent studies have also demonstrated that an HbA1c threshold of 6.0% appropriately discriminates between diabetes diagnosed by OGTT-based methods and nondiabetic subjects [21]. Therefore, our results suggest that the introduction of an HbA1c threshold of 6.0% in screening for diabetes is ideally suited as a screening tool in Japanese subjects.

The analysis of data from Japan showed that a baseline HbA1c of 5.8% or higher imposed a 10-fold increase in the risk of being diagnosed with diabetes over the next 7 years [22]. A large prospective study has demonstrated that an HbA1c cutoff of 5.7% has a sensitivity of 66% and specificity of 88% to identify subjects at increased risk for further progression to diabetes within the next 6 years [23]. Therefore, ADA recommended an HbA1c of 5.7% as a threshold for prediabetes, indicating the need for preventive intervention [16]. The results of the present study showed a modest performance of the HbA1c cutoff value of 5.7% in identifying prediabetes defined by OGTT-based diagnosis with low sensitivity (60.6%), specificity (72.1%), and accuracy (67.6%), which were indicative of a high incidence of false-negative (39.4%) and false-positive (27.9%) results. The high false-negative results of the HbA1c threshold would indicate that a great proportion of prediabetic individuals, who could benefit from lifestyle intervention, would be missed during screening. Therefore, HbA1c is a relatively weak diagnostic tool for detecting prediabetes. Limitations in the use of HbA1c as a diagnostic tool for prediabetes are consistent with those reported by other studies [8–10], particularly when glucose levels are at or near normal values. In the Chinese population, an optimal HbA1c cutoff of 5.7% was determined, with a sensitivity of 59.4% and specificity of 73.9%, for the identification of prediabetes [24]. In the Korean population, an HbA1c cutoff of 5.7% had a sensitivity of 48.6% and specificity of 65.7% for the identification of prediabetes [25]. A systemic review also suggested that the sensitivity of HbA1c for detecting prediabetes was generally low across the studies assessed [23]. Our results suggested that HbA1c was applicable as a screening measure for diabetes, but not for prediabetes.

The strengths of this study are that it is a general population-based study of a specific ethnic group, notable because race and ethnicity may affect HbA1c levels [8]. In addition, the large number of individuals included in the study conferred high statistical power for data analyses. The current study had several limitations. One limitation was that only a single OGTT was performed; however, such an approach is reflective of current clinical practice. Furthermore, all subjects underwent a medical check-up on their own initiative and a considerable number of employees were supported by their companies. Thus, it is possible that these study subjects were likely to be more concerned about their health. It may therefore be difficult to conclude that our study subjects represent the general Japanese population. Our findings must be interpreted accordingly in light of these acknowledged limitations.

## 5. Conclusions

In the Japanese population, optimal HbA1c cutoffs for diagnosing diabetes and prediabetes were 6.0% (sensitivity 83.7%, specificity 87.6%) and 5.7% (sensitivity 60.6%, specificity 72.1%). For the identification of diabetes, HbA1c has optimal sensitivity and specificity to be considered as a mass screening tool. On the other hand, for the identification of prediabetes, HbA1c may be inadequate as a screening tool because of its high false-negative results.

## Figures and Tables

**Table 1:** Demographic and metabolic characteristics of study participants.

	Normal (*n* = 784)	Prediabetes (*n* = 502)	Diabetes (*n* = 86)	*p*
Age (years)	54.5 ± 7.7	57.2 ± 8.5	57.2 ± 7.6	<0.001
Age category (%)				
<50	13.9	8.4	6.0	<0.001
50–59	63.8	57.4	61.6	
≥60	22.3	34.3	31.4	

BMI (kg/m^2^)	22.7 ± 3.0	24.1 ± 3.5	26.3 ± 3.7	<0.001
Waist (cm)	79.9 ± 8.4	84.5 ± 9.4	91.1 ± 9.5	<0.001
SBP (mmHg)	120.9 ± 16.0	127.2 ± 15.4	137.1 ± 16.4	<0.001
DBP (mmHg)	74.2 ± 11.7	77.5 ± 11.2	80.3 ± 11.6	<0.001
UA (mg/dL)	5.4 ± 1.3	5.9 ± 1.3	6.2 ± 1.4	<0.001
eGFR (mL/min./1.73 m^2^)	74.5 ± 11.9	74.2 ± 12.6	74.2 ± 16.3	0.948

Lipids				
TC (mg/dL)	204.2 ± 31.5	204.3 ± 33.5	198.2 ± 34.2	0.248
TG (mg/dL)	109.0 (103.6–114.5)	129.3 (120.3–138.2)	148.3 (129.7–166.8)	<0.001
HDL-C (mg/dL)	60.1 ± 15.1	57.2 ± 13.5	52.9 ± 14.9	<0.001
LDL-C (mg/dL)	118.7 ± 27.3	119.5 ± 29.2	113.6 ± 28.8	0.197

Glucose levels (mg/dL)				
Fasting	91.8 ± 5.7	105.2 ± 8.0	136.9 ± 31.0	<0.001
1h-PG	128.9 ± 36.0	166.1 ± 43.6	223.7 ± 49.2	<0.001
2h-PG	102.3 ± 18.4	131.9 ± 29.9	204.9 ± 50.1	<0.001

HbA1c (%)	5.4 ± 0.3	5.7 ± 0.6	6.7 ± 1.2	<0.001

BMI: body mass index; SBP: systolic blood pressure; DBP: diastolic blood pressure.

UA: uric acid; eGFR: estimated glomerular filtrating ratio.

TC: total cholesterol; TG: triglyceride; HDL-C: HDL-cholesterol; LDL-C: LDL-cholesterol.

PG: postchallenge plasma glucose.

Data are shown as the mean ± standard deviation and percentage (%).

Data of TG are shown as median (interquartile range).

*p* values were calculated using the ANOVA.

**Table 2:** AUCs of ROC in each age category.

	Diabetes	Prediabetes
	AUC (95% CI)	AUC (95% CI)
All ages	0.918 (0.879–0.958)	0.714 (0.685–0.743)
<50 years	0.974 (0.936–1.000)	0.711 (0.622–0.800)
50–59 years	0.908 (0.850–0.965)	0.709 (0.672–0.747)
≥60 years	0.925 (0.865–0.985)	0.702 (0.647–0.758)

AUC: area under the curve; ROC: receiver operating characteristic curve.

CI: confidence interval.

**Table 3:** Sensitivity, specificity, positive and negative predictive values, positive and negative likelihood ratios, and accuracy for detecting diabetes defined by OGTT.

HbA1c (%)	Youden index	Sensitivity (%)	Specificity (%)	Positive predictive value (%)	Negative predictive value (%)	Positive likelihood ratio	Negative likelihood ratio	Accuracy
≧5.6	0.535	94.2	45.7	10.4	99.2	1.7	0.1	48.8
≧5.7	0.625	94.2	59.3	13.3	99.3	2.3	0.1	61.5
≧5.8	0.685	91.9	70.6	17.3	99.2	3.1	0.1	71.9
≧5.9	0.714	88.4	80.2	23.0	99.0	4.5	0.1	80.7
≧**6.0**	**0.725 **	**83.7 **	**87.6 **	**31.2 **	**98.8 **	**6.8**	**0.2**	**87.4 **
≧6.1	0.660	79.1	93.5	38.2	98.5	12.1	0.2	92.6
≧6.2	0.627	69.8	96.3	55.6	97.9	18.7	0.3	94.6
≧6.3	0.557	65.1	97.6	64.4	97.7	27.0	0.4	95.6
≧6.4	0.526	57.0	98.7	74.2	97.2	43.1	0.4	96.1
≧6.5	0.449	53.5	99.1	79.3	97.0	57.3	0.5	96.2

**Table 4:** Sensitivity, specificity, positive and negative predictive values, positive and negative likelihood ratios, and accuracy for detecting prediabetes defined by OGTT.

HbA1c (%)	Sensitivity (%)	Specificity (%)	Youden index	Positive predictive value (%)	Negative predictive value (%)	Positive likelihood ratio	Negative likelihood ratio	Accuracy
≧5.3	92.0	25.4	0.17	44.1	83.3	1.2	0.3	51.4
≧5.4	91.4	26.0	0.17	44.2	82.6	1.2	0.3	51.6
≧5.5	85.5	40.7	0.26	47.9	81.3	1.4	0.4	58.1
≧5.6	71.1	56.5	0.28	51.1	75.3	1.6	0.5	62.2
≧**5.7**	**60.6 **	**72.1 **	**0.33 **	**58.1 **	**74.0 **	**2.2 **	**0.5 **	**67.6 **
≧5.8	49.0	83.2	0.32	65.1	71.8	2.9	0.6	69.8
≧5.9	34.9	89.8	0.25	68.6	68.3	3.4	0.7	68.4
≧6.0	22.5	94.1	0.17	71.1	65.5	3.8	0.8	66.2
≧6.1	13.5	98.0	0.12	81.0	63.9	6.6	0.9	65.0
≧6.2	8.4	99.2	0.08	87.5	62.8	10.9	0.9	63.8
