# Corrigendum to “Optimal Hemoglobin A1c Levels for Screening of Diabetes and Prediabetes in the Japanese Population”

**DOI:** 10.1155/2017/7072538

**Published:** 2017-09-24

**Authors:** Masanori Shimodaira, Shinji Okaniwa, Norinao Hanyu, Tomohiro Nakayama

**Affiliations:** ^1^Department of Internal Medicine, Iida Municipal Hospital, 438 Yawata-machi, Iida, Nagano 395-8502, Japan; ^2^Division of Companion Diagnostics, Department of Pathology of Microbiology, Nihon University School of Medicine, 30-1 Ooyaguchi-kamimachi, Itabashi-ku, Tokyo 173-8610, Japan

In the article titled “Optimal Hemoglobin A1c Levels for Screening of Diabetes and Prediabetes in the Japanese Population” [1], there were errors in Table 3 and Figure 1, which are corrected as follows.

## Figures and Tables

**Figure 1:**
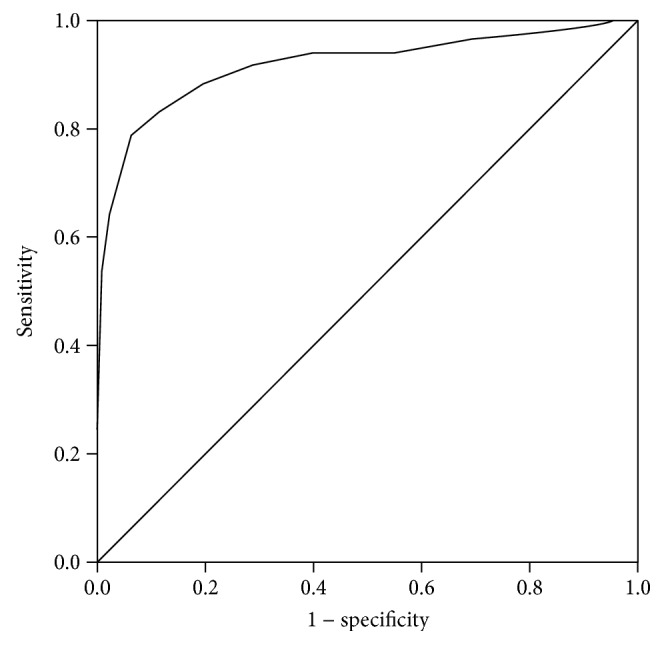
ROC curve analysis for the ability of HbA1c to predict diabetes defined by OGTT values.

**Table 3:** Sensitivity, specificity, positive and negative predictive values, positive and negative likelihood ratios, and accuracy for detecting diabetes defined by OGTT.

HbA1c (%)	Youden's index	Sensitivity (%)	Specificity (%)	Positive predictive value (%)	Negative predictive value (%)	Positive likelihood ratio	Negative likelihood ratio	Accuracy
≧5.6	0.399	94.2	45.7	10.4	99.2	1.7	0.1	48.8
≧5.7	0.535	94.2	59.3	13.3	99.3	2.3	0.1	61.5
≧5.8	0.625	91.9	70.6	17.3	99.2	3.1	0.1	71.9
≧5.9	0.685	88.4	80.2	23.0	99.0	4.5	0.1	80.7
**≧6.0**	**0.714**	**83.7**	**87.6**	**31.2**	**98.8**	**6.8**	**0.2**	**87.4**
≧6.1	0.660	69.8	96.3	55.6	97.9	18.7	0.3	94.6
≧6.2	0.627	65.1	97.6	64.4	97.7	27.0	0.4	95.6
≧6.3	0.557	57.0	98.7	74.2	97.2	43.1	0.4	96.1
≧6.4	0.526	53.5	99.1	79.3	97.0	57.3	0.5	96.2
≧6.5	0.449	45.3	99.5	86.7	96.5	90.6	0.5	96.1
